# Case Report: Analysis of the missed diagnosis of atrial septal defect with partial pulmonary vein dual drainage

**DOI:** 10.3389/fcvm.2026.1778760

**Published:** 2026-03-26

**Authors:** Chi-heng Zhou, Yuan-yuan Zhang, Qun-shan Shen

**Affiliations:** Congenital Heart Disease Center, Wuhan Asia Heart Hospital, Wuhan, China

**Keywords:** atrial septal defect, cardiac catheterization, cardiac CT, partial anomalous pulmonary venous connections, transthoracic echocardiography

## Abstract

**Objective:**

This study aimed to investigate the reasons for the misdiagnosis of a case of atrial septal defect (ASD) complicated by partial anomalous pulmonary venous connection (PAPVC) to improve clinical diagnosis accuracy in similar cases.

**Methods:**

The clinical data, imaging findings, and surgical records of a patient with ASD complicated by PAPVC in our department were retrospectively analyzed, and the reasons for the missed diagnosis were comprehensively analyzed in combination with the literature.

**Results:**

The patient was diagnosed with PAPVC at another hospital; however, neither PAPVC nor ASD was identified on transthoracic echocardiography at our hospital. Cardiac CT and pulmonary angiography showed that the right upper pulmonary vein communicated with both the superior vena cava and the left atrium at the same time; however, the presence of an ASD was still not detected. An upper cavity ASD was found during thoracoscopic orthodontic surgery for downward PAPVC.

**Conclusion:**

The diagnosis of superior vena cava ASD associated with anomalous pulmonary vein drainage is prone to missed diagnosis, and when the pulmonary vein drains into both the superior vena cava and the left atrium at the same time, it increases the difficulty of detecting PAPAC by echocardiography. During transesophageal echocardiography examination, the integrity of the atrial septum should be repeatedly assessed in multiple sections, and continuous tracking of the pulmonary veins can help reduce the risk of missed diagnoses in similar cases. In addition, surgeons should fully communicate with echocardiographers and imaging specialists before surgery to formulate an individualized surgical plan.

## Introduction

Atrial septal defects (ASDs) are classified into several types, including primary foramen, secondary foramen, venous sinus (upper and lower cavum), coronary sinus, compound type, single atrium, ethmoid foramen, and patent foramen ovale. These defects can exist alone or in association with other malformations (1). Partial anomalous pulmonary venous connection refers to a condition in which one to three pulmonary veins fail to connect to the left atrium and instead are connected to the right atrium or a systemic vein (2). An atrial septal defect causes an atrial horizontal left-to-right shunt, while anomalous pulmonary vein drainage can also increase the right heart volume load. Both of them impose a great burden on the right heart of the patient. Early diagnosis and timely treatment can significantly improve the prognosis of patients. In the diagnosis of right-sided anomalous pulmonary venous drainage associated with sinus venosus atrial septal defect using transthoracic echocardiography, the signal of atrial horizontal shunt is often unclear due to the influence of blood flow in the upper and lower cavities on the parasternal section, which is easy to be missed and misdiagnosed (3). This paper reports a case of sinus venosus atrial septal defect complicated by partial anomalous pulmonary venous connection (PAPVC) that was initially missed, with the aim of providing a reference for similar clinical cases.

## Case introduction

The patient, a 43-year-old woman, presented to a local hospital with complaints of “left facial paresthesia.” Brain MRI and intracranial MRA showed no evidence of acute cerebral infarction and obvious abnormalities in the intracranial blood vessels in the pons and left pons. Contrast-enhanced echocardiography demonstrated a large number of contrast agent microbubbles in the left heart at rest. After the Valsalva maneuver, a large number of contrast agent microbubbles echo in the left heart, suggesting “cerebral artery occlusion with cerebral infarction.”

**Figure 1:**
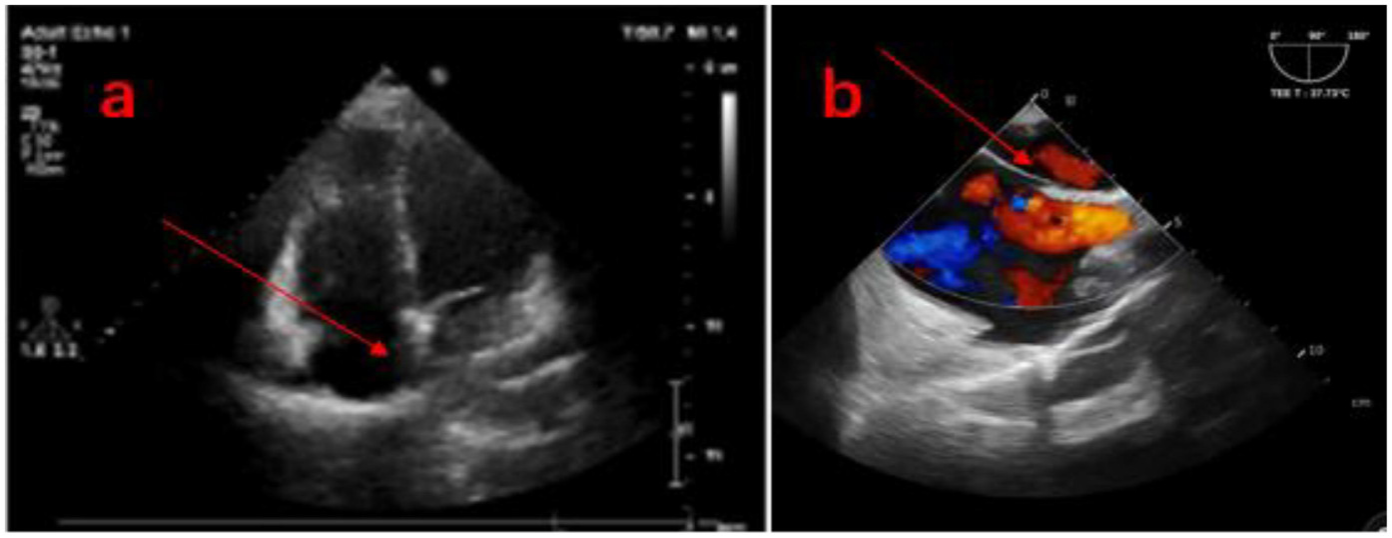
(**a**) The atrial septum is complete on the four-chamber view of TTE, (**b**) the atrial septum is complete on the two-chamber view of TEE. The arrow in the picture shows the complete room partition.

**Figure 2:**
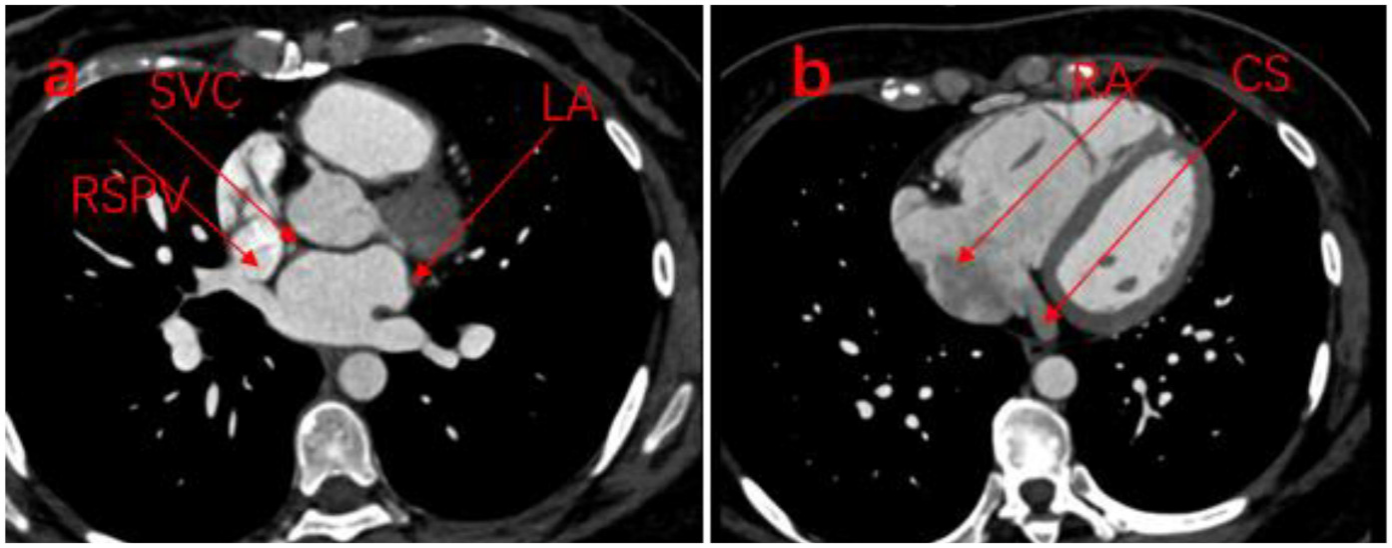
**(a)** Cardiac CT showing that the lateral wall of the RSPV communicates with the SVC and still drains back into the LA. **(b)** Cardiac CT showing that an intact coronary sinus with normal drainage into the reft atrium. RSPV, right superior pulmonary vein; SVC, superior vena cava; LA, left atrium; CS, coronary sinus.

**Figure 3:**
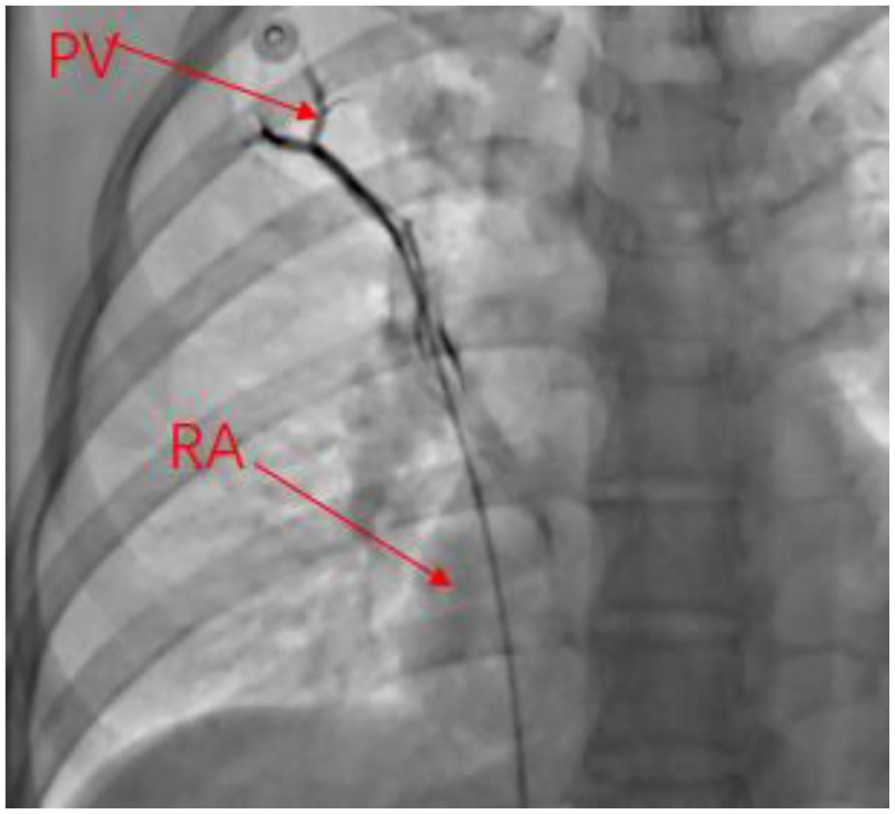
Right cardiac catheterization showing that the RSPV was explored through the SVC, and the contrast agent returned to the LA during selective pulmonary venography. PV, pulmonary vein; RA, right atrium.

Transthoracic echocardiography (TTE) showed anomalous pulmonary vein drainage and pulmonary hypertension, and the patient was subsequently referred to our hospital. TTE after admission showed that the ventricular septum was slightly thick and the tricuspid valve was slightly regurgitated (the echo of the atrial septum was continuous and complete; Figure 1a). The results of transesophageal echocardiography (TEE) are consistent with those of transthoracic echocardiography (Figure 1b). Cardiac CT showed that the pulmonary artery and its branches were dilated, along with partial anomalous pulmonary vein drainage (the proximal side wall of the right upper pulmonary vein was found to communicate with the superior vena cava, while four pulmonary vein orifices were observed to drain into the left atrium; Figure 2). Right cardiac catheterization showed a pulmonary-to-systemic blood flow ratio (Q_P_/Q_S_) of 2.0, and pulmonary angiography further confirmed communication between the superior vena cava and the right upper pulmonary vein (Figure 3). A transcranial Doppler ultrasound bubble test was performed, monitoring the single channel (left middle cerebral artery) at dual depths (48 mm/60 mm); after injection of agitated saline, 11–25 microembolic signals were detected within 25 s at rest, and after the Valsalva maneuver, a large number of microembolic signals were observed within 25 s. Laboratory examination showed that NT-proBNP levels, liver function, renal function, myocardial enzyme spectrum, electrolytes, and blood routine were all within normal limits.

**Figure 4:**
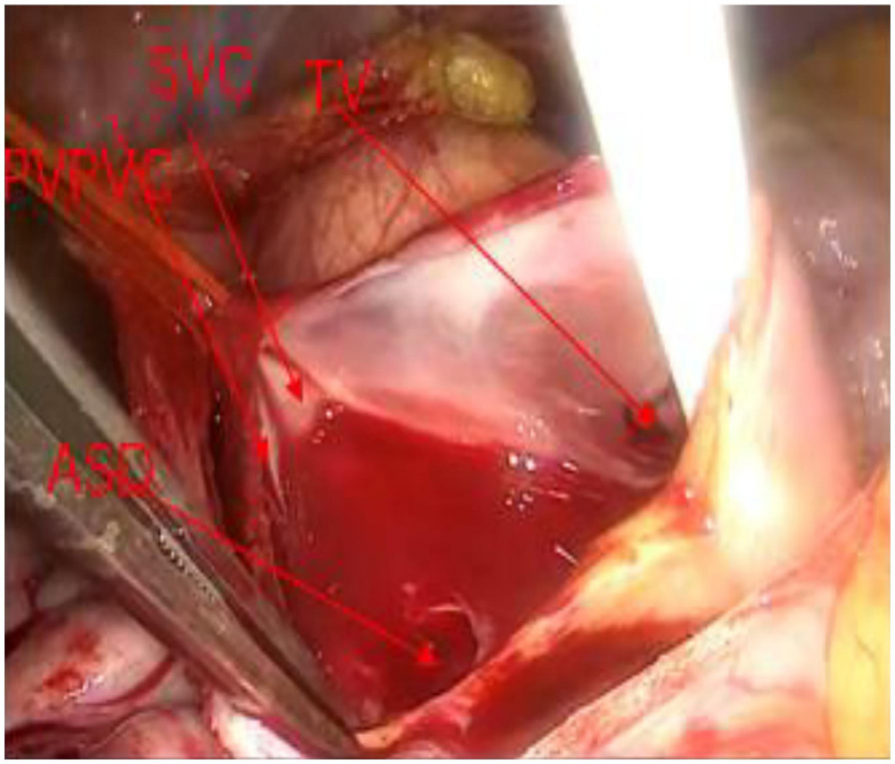
After opening the RA during surgery, the RSPV was observed to communicate with the SVC, and a 2-cm defect was identified in the atrial septum. TV, tricuspid valve.

After thorough discussion with the patient and her family, on the eighth day after hospitalization, the patient underwent thoracoscopic repair of the partial anomalous pulmonary venous drainage. A 5-cm incision was made along the right anterior axillary line at the fourth intercostal space, and a thoracoscope was placed in the fifth intercostal space. Extracorporeal circulation was established through the femoral artery and jugular vein, and the superior and inferior vena cava were ligated through the chest wall incision, blocking the superior and inferior vena cava without cooling and beating. A longitudinal incision was made along the superior vena cava, extending downward to fully expose the superior vena cava and the right atrium. During the surgery, it was observed that the right and middle pulmonary veins drained into the superior vena cava and the right atrium, respectively, with a defect measuring 2 cm (Figure 4). An autologous pericardial patch was used to repair the atrial septal defect and redirect the right upper and middle pulmonary veins into the left atrium. A bovine pericardial patch was applied to widen the superior vena cava and close the right atrial incision. Intraoperative esophageal echocardiography confirmed the absence of residual shunt at the atrial septum, and the blood flow velocity in the superior vena cava was not fast. Postoperatively, symptomatic treatment, such as ventilator-assisted breathing, anti-infective therapy, myocardial nutrition, and maintenance of internal environment balance, was continued in the intensive care unit. After 3 days in the intensive care unit, her condition stabilized, and she was transferred to the general ward, where she continued to receive supportive treatment, including anti-infective therapy and maintenance of cardiac function and internal environment balance. He was discharged on the 11th day after the operation.

## Discussion

This paper reports a case of PAPVC that was diagnosed preoperatively and was found to be complicated by a superior atrial septal defect during surgery. The patient was referred to our hospital after being diagnosed with PAPVC at another hospital following a cerebral infarction. Neither ASD nor PAPVC was identified by transthoracic echocardiography and transesophageal echocardiography at our hospital. Cardiac CT showed that the proximal side wall of the right upper pulmonary vein communicated with the superior vena cava while still draining into the left atrium; consequently, four pulmonary veins were seen in the left atrium on echocardiography, which contributed to the missed diagnosis of PAPVC by echocardiography. During surgery, it was found that the ASD was located at the entrance of the superior vena cava, which was connected to the right upper pulmonary vein; therefore, echocardiography mistakenly regarded this ASD as part of the superior vena cava. Echocardiography remains the first choice for the clinical diagnosis of congenital heart disease because it is non-invasive, cost-effective, and convenient. The pulmonary veins are relatively distant from the acoustic window, and the hemodynamic changes are not obvious in cases of dual drainage of the PAPVC into the superior vena cava and the LA, which is one of the reasons for missed diagnosis (4). In addition, sonographers mostly rely on the number of pulmonary veins entering the LA as the sole criterion for PAPVC diagnosis, without continuous tracking of the pulmonary veins, which is another reason for missed diagnosis, similar to previously reported cases of PAPVC complicated by a pulmonary venous variation (5). Although this patient did not have a pulmonary vein variation, the right upper pulmonary vein communicated with both the superior vena cava and the LA at the same time, allowing four pulmonary veins to be visualized in the LA, consistent with the case reported by Tanaka et al. (6). Finally, the dual communication of the right upper pulmonary vein was confirmed by pulmonary venography and cardiac CT. A retrospective analysis shows that the concordance rate between ultrasound findings and surgical and cardiac CT diagnosis results is only 93.3% (7). Therefore, in the diagnosis of pulmonary vein-related diseases, ultrasound examination should incorporate different imaging positions and examination sites beyond routine sections. A systematic approach combining continuous pulmonary vein tracking with focused assessment of key sections may help reduce the rate of missed diagnoses.

The surgical indications for single anomalous pulmonary vein drainage remain controversial (8), and overall clinical cardiac function, right ventricular performance, and the pulmonary–systemic circulation flow ratio need to be evaluated in detail preoperatively [Q_P_/Q_S_ >1.5 is considered an indication for surgical intervention ] (9). Accordingly, right cardiac catheterization was performed before surgery. Given the inconsistency between echocardiographic and cardiac CT results, the guidewire was advanced into the superior vena cava through the right atrium during cardiac catheterization, and the pulmonary vein was explored in the superior vena cava. Finally, the guidewire was advanced into the right pulmonary vein via the superior vena cava. During pulmonary venography, the contrast agent flowed back into the left atrium; however, the superior vena cava and the right atrium were not imaged. Afterward, combined with the intraoperative findings, the cardiac catheterization examiner recalled that the guidewire may have reached the pulmonary vein through the atrial septal defect while being advanced from the right atrium toward the superior vena cava; as a result, the contrast agent returned to the left atrium during selective pulmonary venography, without opacifying the superior vena cava and the right atrium.

**Figure 5:**
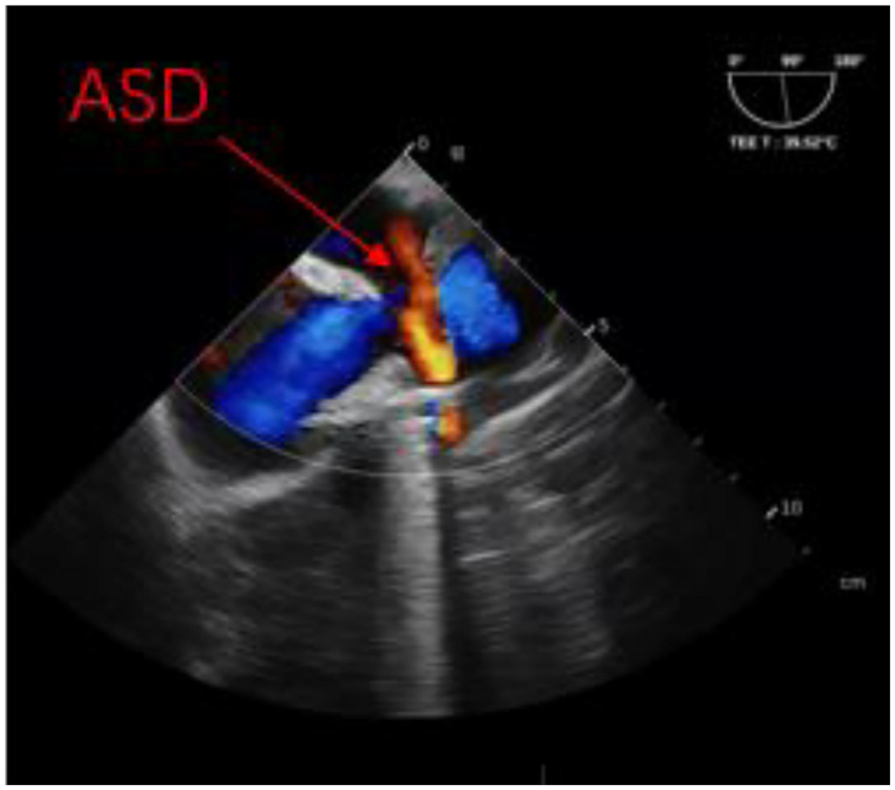
Non-standard double-lumen view of TEE and superior atrial septal defect.

Intraoperative exploration of this patient showed that the ASD was located at the superior vena cava, close to the right upper pulmonary vein and communicating with the superior vena cava; consequently, preoperative TTE and TEE revealed an intact atrial septum and misinterpreted the ASD as a part of the superior vena cava. Previous reports on ASD complicated by PAPVC (10, 11) have shown that transthoracic echocardiography has 100% specificity, and 20% of missed diagnoses can be identified by transesophageal three-dimensional echocardiography (12). One limitation of this case is that a preoperative three-dimensional ultrasound was not performed. In addition, the patient's heart did not stop during surgery, which may have limited the clarity of the intraoperative anatomical structure and represents another limitation of this case. Based on the special circumstances of this case, our hospital has since explored atrial septal defects in multiple sections during TEE examination in patients with similar cardiac CT and TTE results and successfully screened an atrial septal defect of the superior chamber (Figure 5).

To sum up, it is still difficult to diagnose various types of ASDs solely by echocardiography and imaging. When echocardiographic findings are inconsistent with cardiac CT reults, repeat TEE examination should be performed, and atrial septum integrity should be repeatedly explored in multiple sections (including but not limited to four-chamber, double-chamber, and five-chamber views); if uncertainty persists, it can be further clarified by other diagnostic methods, such as whether the superior vena cava and atrial septum are combined with anomalous pulmonary venous drainage and the ASD during cardiac catheterization, as well as timely communication with experienced surgeons and combining with rich experience in previous operations in preoperative simulation. This article aims to provide a reference for similar clinical cases.

## Data Availability

The original contributions presented in the study are included in the article/Supplementary Material, further inquiries can be directed to the corresponding author.
